# Machine learning approach informs biology of cancer drug response

**DOI:** 10.1186/s12859-022-04720-z

**Published:** 2022-05-17

**Authors:** Eliot Y. Zhu, Adam J. Dupuy

**Affiliations:** 1 Department of Anatomy and Cell Biology, The University of Iowa, Iowa City, IA USA; 2 Holden Comprehensive Cancer Center, The University of Iowa, Iowa City, IA USA; 3 Cancer Biology Graduate Program, The University of Iowa, Iowa City, IA USA; 4 The Medical Scientist Training Program, The University of Iowa, Iowa City, IA USA

**Keywords:** Drug resistance, Machine learning, Paclitaxel, Ovarian, PAX8, MECOM, AKT

## Abstract

**Background:**

The mechanism of action for most cancer drugs is not clear. Large-scale pharmacogenomic cancer cell line datasets offer a rich resource to obtain this knowledge. Here, we present an analysis strategy for revealing biological pathways that contribute to drug response using publicly available pharmacogenomic cancer cell line datasets.

**Methods:**

We present a custom machine-learning based approach for identifying biological pathways involved in cancer drug response. We test the utility of our approach with a pan-cancer analysis of ML210, an inhibitor of GPX4, and a melanoma-focused analysis of inhibitors of *BRAF*^*V600*^. We apply our approach to reveal determinants of drug resistance to microtubule inhibitors.

**Results:**

Our method implicated lipid metabolism and Rac1/cytoskeleton signaling in the context of ML210 and BRAF inhibitor response, respectively. These findings are consistent with current knowledge of how these drugs work. For microtubule inhibitors, our approach implicated Notch and Akt signaling as pathways that associated with response.

**Conclusions:**

Our results demonstrate the utility of combining informed feature selection and machine learning algorithms in understanding cancer drug response.

**Supplementary Information:**

The online version contains supplementary material available at 10.1186/s12859-022-04720-z.

## Background

Drug resistance and off-target toxicity are two major obstacles for precision cancer treatment. Experimental approaches to understand these areas of research depend on the use of genetic screens or drug perturbation experiments paired with -omics profiling. However, such experiments require large commitments of resources including cell culture, genetic screening constructs, sequencing costs, and personnel. Analysis of publicly available pharmacogenomic datasets is a vastly less expensive option to understand the biology of cancer drugs. The difficulty with using in silico approaches is that meaningful signals may be weak and not easily detectable. Considering this challenge, Machine learning (ML) algorithms has become an increasingly popular strategy to build predictive models that utilize molecular patients of tumor or cancer cells to predict and understand patients’ or cell lines’ response to drugs [1–11].

Existing strategies for building drug response classifiers are incredibly diverse, utilizing various combinations of inputs, feature selection approaches, and algorithms. Here, we built a machine learning algorithm focused on informing the biological processes that drive cancer drug response. We do so by integrating prior knowledge of biological pathways and protein–protein interaction data. We tested our approach on two compounds: ML210, a selective covalent inhibitor of glutathione peroxidase 4 (GPX4) and the selective BRAF^V600^ inhibitors vemurafenib (VEM) and dabrafenib. We also used our approach to identify pathways that inform response to anti-tubulin drugs.

## Methods

All analysis was performed in R using custom scripts. First, consider KEGG pathways belonging to Metabolism, Genetic Information Processing, Environmental Information Processing, and Cellular Processes. This list contains ~ 150 pathways. For each pathway, compute the pathway activity scores. The pathway activity score is defined as the t-score of the pathway activities across drug-sensitive and drug-resistant cell lines. Specifically, the pathway activity for pathway *p*, sample *j,* and gene *i,* is given by,

$$a_{pj} = \mathop \sum \limits_{i = 1}^{k} \frac{{z_{ij} }}{\sqrt k }$$

where *z* is the normalized gene expression. The number of genes to use for each pathway, or *k,* is determined using a greedy search strategy. That is, compute the t-score for each gene for a given pathway. Rank genes in increasing order if average t-scores are less than zero or in decreasing order, otherwise. Iterate over *i* until the maximum $a_{p}$ is found. In other words, *k* is the smallest number that maximizes the t-score for $a_{p}$. See [12] for complete details on computing the pathway activity score.

For the BRAFi analysis, pathways are determined to be significant using a null distribution generated by permuting the cell line labels. For the ML210 and Paclitaxel analysis, pathways with pathway activity scores within the bottom or top 10th or 20th percentiles, respectively, were retained for further analysis. Significance thresholds were designed to return ~ 20% of the initial number of input KEGG pathways.

Next, take all genes from the pathways deemed significant. Bin these genes into mutually exclusive network modules. Genes are grouped together into mutually exclusive network modules through hierarchical clustering of the dissimilarity between genes. Dissimilarity is computed as 1 minus the standard topological overlap measure described in [13]. The adjacency matrix used to compute the topological overlap was derived using STRING protein–protein interactions [14]. Namely, we considered an edge to exist between two genes if they had a STRING combined score of ≥ 0.4.

Then, determine the most informative genes in each module, separately, using Boruta, a random-forest-based feature selection algorithm, with default parameters [15]. Genes with a finalDecision of “Confirmed” was retained for further analysis. Boruta determines variable importance by comparing the performance of an attribute releative to permutated versions of it within random forest classification.

Finally, take all the informative genes from the previous step and build a classifier using the support vector machine learning algorithm with recursive feature elimination (RFE). We used the implementation provided at https://github.com/johncolby/SVM-RFE. RFE involves running the SVM iteratively while removing the least informative feature at each iteration. The rank of the feature is inversely related to the iteration it was removed by the SVM algorithm. For our analysis, the rank for a feature is given as an average of a feature’s rank across ten-fold cross validation for the ML210 and Paclitaxel analysis or leave-one-out-cross validation for the BRAFi analysis. The ranking of each feature determines the importance of the module it belongs to. The biological representation of each module was determined using  Gene Ontology pathways enrichment analysis implemented by the limma R package [16].

To perform our machine learning analysis, we used RMA-normalized microarray gene expression from Genomics of Drug Sensitivity in Cancer (GDSC). We used ML210 and PTX drug response data from the Cancer Therapeutics Response Portal V2 (CTRP v2). We used VEM and Dabrafenib response data from GDSC. We used area under the curve (AUC) as the metric for drug response. The cutoff for ML210 resistance was set at an AUC of 9, which qualitatively separated two modes of the AUC distribution (Additional file 1: Figure S1). The cutoff for PTX resistance was set at 5 to distinguish the most sensitive cancers. The cutoff for drug response for BRAF inhibition was set at the 5^th^ percentile of the AUC for VEM or Dabrafenib in the GDSC. Two BRAF inhibitors were used to compensate for missing data.

The *singscore* [17] R-package was used to compute the pathway enrichment scores for the 4-gene NOTCH3/PAX8 across ovarian cancer cell lines. The *biomaRt* R-package was used for data wrangling [18]. The *ggplot2* R-package was used for visualization [19].

For the t-test analysis, genes that had a Holm-Bonferroni corrected *p*-value of < 0.1 were deemed as significant. Cell lines were labeled as sensitive or resistant to a drug of interest as described for each case study. Elastic net regression was performed using glmnet and caret R packages [20, 21]. AUCs for the respective drugs were regressed on the gene expression of the top 5000 most variably expressed genes. The optimal lambda was selected using ten-fold cross validation on models using different parameters determined by tuneLength = 20. Genes with non-zero coefficients were used for enrichment analysis.

## Results

### Design and conceptualization

**Fig. 1:**
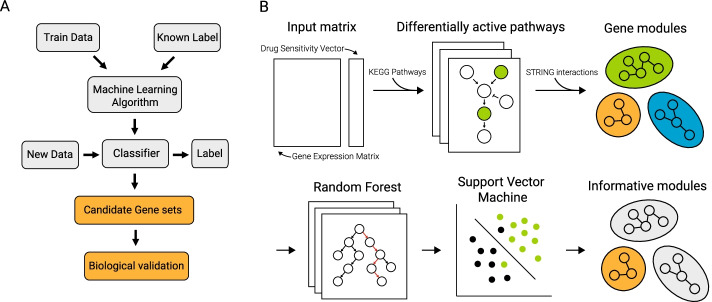
Workflow of machine learning analysis. **A** Schematic that emphasizes the goal of our analysis compared to that of typical workflows. **B** Schematic of our mechanism-driven machine learning approach

We constructed a supervised learning algorithm to nominate biological processes that underlie cancer drug response. Our approach emphasizes prioritization of biologically meaningful features used for classification rather than predictive performance (Fig. 1). We trained our algorithm using only gene expression and drug sensitivity data. We opted to only used gene expression as this data type consistently performed the best as a standalone dataset in a metanalysis of the 44 machine learning algorithms submitted to the NCI-DREAM drug sensitivity prediction challenge [22]. We also favored gene expression as it is known that transcriptomic diversity better explains phenotypic heterogeneity in some cancers, such as cutaneous melanoma [23].

Conceptually, our approach is based on the support vector machine learning algorithm combined with multiple layers of feature selection. Additionally, we use protein–protein interaction data to annotate important features with pathway-level information. Ultimately, our approach returns a ranked list of features, *i.e.* genes, that are grouped into mutually exclusive modules containing closely interacting genes. This strategy enables ranking of known biological processes like pathway enrichment analysis but requires much fewer informative, or differentially expressed, genes.

### Case Study 1: Pathways that inform GPX4i sensitivity

**Fig. 2:**
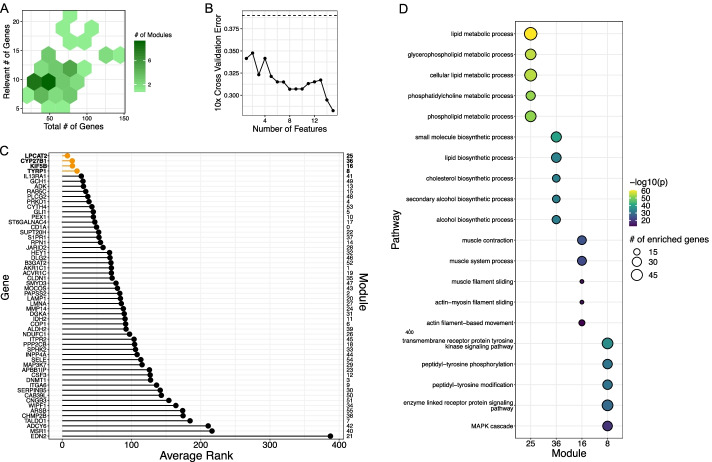
Pathways that inform ML210 response. **A** Visualization of gene distribution within modules. Relevant genes are those that passed the Boruta filtering step. X-axis denote total number genes per module, y-axis denotes number of relevant genes, and the shading indicates total number of modules. **B** Ten-fold  cross validation error as a function of number of features used in the SVM model. Dashed line indicates no information rate, i.e. the error made if the class with the greatest frequency was selected. **C** Minimum feature ranking for each module. **D** GO Biological Processes pathway enrichment of genes contained within modules presented in C). P-values shown are corrected for multiple hypothesis testing using the Holm-Bonferroni method

ML210 was initially discovered in a high-throughput screening effort as an agent that was selective against HRAS-driven oncogenesis in fibroblasts [24]. However, ML210’s mechanism of action was unknown at the time of its discovery. Later, it was found that ML210 kills cells via induction of ferroptosis through inhibition of GPX4 [25, 26]. We applied our approach on all cancer cell lines with gene expression and drug response data to ML210. Pathway activity feature selection returned pathways listed in Additional file 3: Table S1. This selection step retained 2439 genes. Boruta feature selection returned genes that enriched for GO Biological processes in Additional file 4: Table S2. This selection step retained 395 genes across 39 modules. Our method ranked lipid metabolism as the top pathway that determines sensitivity to ML210 (Fig. 2). This result is consistent with the knowledge that the balance of monounsaturated fatty acids (MUFAs) and polyunsaturated fatty acids (PUFAs) determines susceptibility to ferroptosis [27, 28]. As a negative control for the utility of our method, we performed enrichment analysis using genes determined to be significant using t-test or those retained by elastic net (Additional file 5: Table S3, Additional file 6: Table S4). The top results from our approach did not overlap with that of the standard analysis we tried.

The first step of the proposed approach is to input a set of KEGG pathways. As described in the methods, we performed this analysis using KEGG pathways belonging to Metabolism, Genetic Information Processing, Environmental Information Processing, and Cellular Processes. To test what would happen if all pathways were included, we repeated the analysis for ML210 using all human KEGG pathways (Additional file 2: Figure S2). Lipid metabolism and actin cytoskeleton pathways remained top candidates, but the other two top pathways changed.

### Case Study 2: Pathways that inform BRAFi sensitivity

**Fig. 3:**
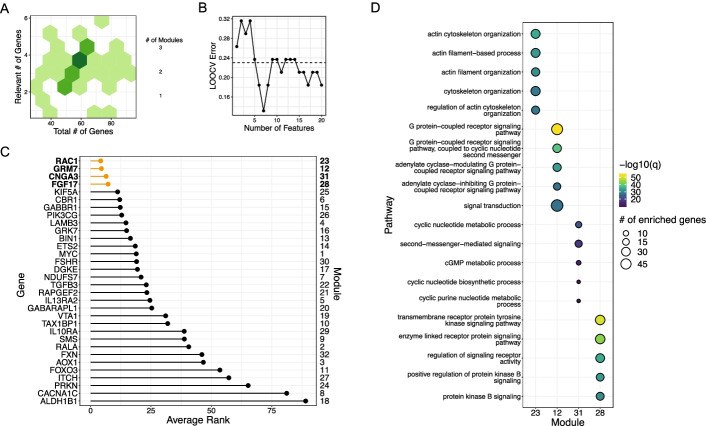
Pathways that inform BRAFi response. **A** Visualization of gene distribution within modules. Relevant genes are those that passed the Boruta filtering step. X-axis denote total number genes per module, y-axis denotes number of relevant genes, and the shading indicates total number of modules. **B** Leave-one-out cross validation error as a function of number of features used in the SVM model. Dashed line indicates no information rate, i.e. the error made if the class with the greatest frequency was selected. **C** Minimum feature ranking for each module. **D** GO Biological Processes pathway enrichment of genes contained within modules presented in **C**). *P*-values shown are corrected for multiple hypothesis testing using the Holm-Bonferroni method

Next, we tested our approach on selective inhibitors of *BRAF*^*V600E*^. We analyzed only cutaneous melanoma cell lines with the *BRAF*^*V600E*^ mutation, which is present in ~ 50% of this type of cancer. Even when this mutation is present, drug response to BRAF inhibitors is heterogenous with some melanomas more resistant to BRAF inhibition (BRAFi) than others. Pathway activity feature selection returned pathways listed in Additional file 7: Table S5. This selection step retained 3223 genes. Boruta feature selection returned genes that enriched for GO Biological processes in Additional file 8: Table S6. This selection step retained 169 genes across 36 modules. For the BRAF inhibitors, our method identified Rac1/cytoskeletal signaling as the most salient driver of drug resistance (Fig. 3). As a negative control for the utility of our method, we performed enrichment analysis using genes determined to be significant using t-test or those retained by elastic net (Additional file 9: Table S7, Additional file 10: Table S8). We found that Actin/cytoskeleton processes were highly ranked by our approach but not by the t-test nor elastic net. However, both our approach and the p-value strategy prioritized the “transmembrane receptor protein tyrosine kinase signaling pathway.” This finding is consistent with other studies that report certain RTKs such as PDGFRB and CSF1R drive intrinsic drug resistance to BRAFi in *BRAF*^V600^ cutaneous melanoma [29, 30].

### Case Study 3: Pathways that inform sensitivity to anti-tubulin drugs

**Fig. 4:**
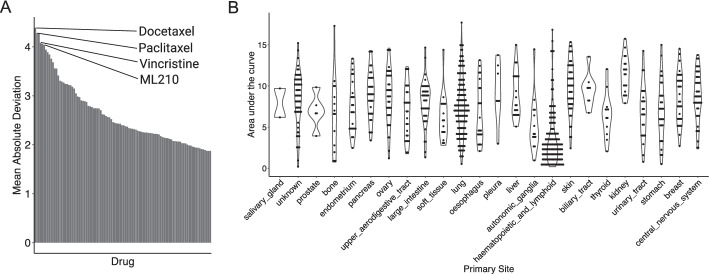
Paclitaxel exploratory analysis. **A** Variability of response to drugs in the CTRPv2 database. **B** Response to PTX across cell lines of different cancer types contained in CCLE. Higher AUC means more resistant to drug

For our last case study, we wondered if our approach could identify new insights for drugs where the mechanisms of response are less understood. We took an -omics approach and looked for drugs with heterogeneous response. To this end, we ranked drugs in CTRPv2 with respect to the mean absolute deviation of the AUC. In addition to ML210 discussed previously, three anti-tubulin drugs (paclitaxel (PTX), docetaxel, vincristine) were among those with the most variable response (Fig. 4A). Sensitivity to anti-tubulin drugs were highly correlated (Pearson correlation of 0.83 for paclitaxel and vincristine, 0.92 for paclitaxel and docetaxel, and 0.83 for vincristine and docetaxel), suggesting similar mechanisms of action. Pan-cancer analysis of response to paclitaxel shows that hematopoietic cancers are generally more sensitive to microtubule disruption. However, response within cancers of other sites, e.g. lung, ovary, was also heterogeneous (Fig. 4B).

**Fig. 5:**
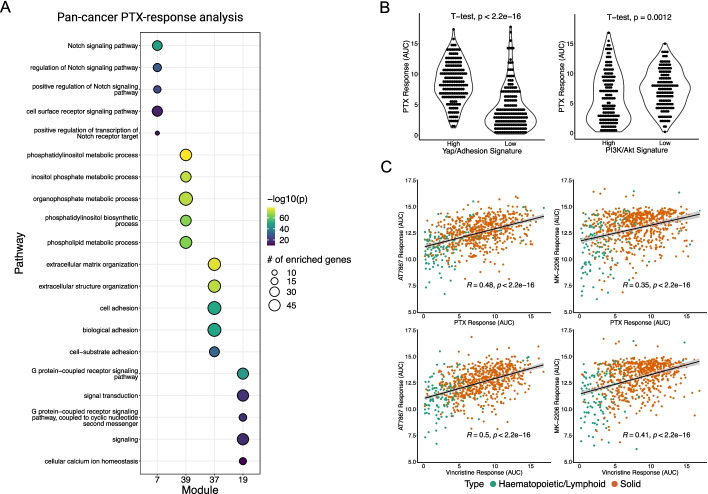
Pan-cancer analysis of pathways that inform paclitaxel response. **A** GO Biological Processes pathway enrichment of genes contained within the top predictive modules. **B** PTX response in cancer cell lines separated by Yap/Adhesion gene signature (left) or PI3K/Akt gene signature (right). Higher AUC means more resistant to drug. **C** Correlation of microtubule inhibitors with Akt inhibitors

In the era of precision oncology, anti-tubulin drugs are considered “non-targeted”, but unexpectedly we observed that the response to anti-tubulin drugs was highly disparate across different cancer cell lines. This suggests that there may be cancer cell intrinsic features that dictate sensitivity to these drugs. To explain this variation, we applied our analysis approach on PTX. Pathway activity feature selection retained 3232 genes. Boruta feature selection retained 822 genes across 49 modules. Pan-cancer analysis suggested that Notch, Akt, and adhesion signaling may be involved in PTX-response (Fig. 5A). Notch signaling likely was used as a predictor because Notch is a critical driver of hematopoietic cancers, which happen to be generally sensitive to PTX-inhibition. As a negative control for the utility of our method, we performed enrichment analysis using genes determined to be significant using t-test or those retained by elastic net (Additional file 11: Table S9, Additional file 12: Table S10).

To confirm the relevancy of cell adhesion and Akt signaling, we computed previously published gene signatures for these pathways and tested whether the response to PTX was different between cell lines with high/low cell adhesion or Akt signaling signatures [31, 32]. Cell adhesion signaling is known to be regulated by Yap/TEADs, and in general, cancer cells can be classified into Yap^on^ or Yap^off^ cancers [33]. Using a gene signature based on genes elevated in Yap^on^ cancers, we found that cancers with low Yap signature was more sensitive to PTX inhibition. Conversely, we found that cancer cell lines that had a high PI3K/AKT signature tended to more sensitive to PTX (Fig. 5B). As there are several targeted inhibitors of Akt, we further investigated the connection between PTX sensitivity and PI3K/AKT signaling by computing the correlation between PTX and Vincristine response with two different pan-Akt inhibitors (AT7867, MK2206). We observed statistically significant correlations between the response to microtubule and Akt inhibitors (Fig. 5C).

**Fig. 6:**
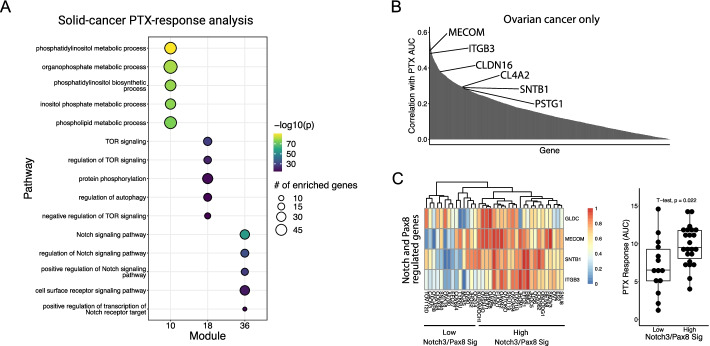
Solid-cancer analysis of pathways that inform paclitaxel response. **A** GO Biological Processes pathway enrichment of genes contained within the top predictive modules. **B** Correlation of PTX response with expression of genes in ovarian cancer cell lines. **C** Max–min normalized gene expression of NOTCH3/PAX8 genes in ovarian cancer cell lines (left). Response to PTX in ovarian cancer cell lines separated by the expression of four genes shown in the heatmap on the left (right). Higher AUC means more resistant to drug

Since haemopoietic cancers have unique signaling features, *i.e*. Yap^off^ and Notch^hi^, and contribute to a large percentage of PTX-sensitive samples, we performed the same analysis wherein we only used solid tumor cell lines. Pathway activity feature selection retained 2667 genes. Boruta feature selection retained 223 genes across 43 modules. Surprisingly, even when we excluded blood cancers, Notch signaling remained a predictor of response to PTX, along with Akt signaling (Fig. 6A). As a negative control for the utility of method we performed enrichment analysis using genes determined to be significant using t-test or those retained by elastic net (Additional file 13: Table S11, Additional file 14: S12). To confirm the connection between Notch and PTX response, we narrowed our focus on ovarian cancer, where PTX remains a standard of care. To get a general view of pathways associated with PTX-resistance, we identified genes expressed in ovarian cancer cell lines that were highly correlated with PTX response (Fig. 6B). Of note, one of these genes was MECOM. The locus at chromosome 3q21 contains MECOM and encodes the MDS1 and EVI1 proteins, under the control of two separate promoters. These proteins have been implicated in leukemia development [34–36].

Recently, it was shown that MECOM interacts with PAX8, a transcription factor that is an oncogene for ovarian and kidney cancers and can serve as an indicator of PAX8 transcriptional activity [37]. To determine the relationship with Notch signaling, we analyzed a published dataset where NOTCH3 was overexpressed in a murine ovarian surface epithelial cell line [38]. Interestingly, in this model, overexpression of NOTCH3 resulted in a four-fold increase in MECOM. In support of the connection between Notch and PAX8 signaling, we found that other genes positively regulated by NOTCH3 (> four-fold increase upon NOTCH3 overexpression), including NGLDC, SNTB1, and ITGB3, belonged to a the 29-gene PAX8 signature that was reduced upon PAX8 knockdown in multiple human ovarian cancer cell lines [37]. Profiling PTX response using a four-gene signature derived only from the NOTCH3 and PAX8 regulated genes, we observe that ovarian cell lines from CCLE with high NOTCH3/PAX8 transcriptional signature were more resistant to PTX (Fig. 6C). This observation suggests a previously unreported connection between drug resistance to PTX and NOTCH3/PAX8 signaling.

## Discussion

Machine learning approaches for modeling cancer drug response have shown promise in predicting cancer drug sensitivity but may not inform biological processes that underlie response. Existing strategies used to reveal this information include pathway enrichment on highly weighted genes prior to the first hidden layer in a deep neural network, that obtained from models such as decision trees, or those with high Shapley values of deep neural networks [9, 39–41]. In this study, we extract biological meaning from a machine learning model by combining multiple layers of feature selection with a ranking process performed through the support vector machine. Furthermore, instead of using all available genes, we only utilize genes that fall within curated pathways and group such genes within interacting modules—sacrificing performance for interpretability. We demonstrate the utility of our approach with three test-cases. For each case, we also confirmed that standard analyses did not prioritize the same pathways that our approach did. Namely, we computed enriched pathways in genes that were differentially expressed between sensitive and drug resistant cell lines using the t-test. We also computed enriched pathways in genes, selected by elastic net, that could best model drug response.

Our knowledge-guided machine learning analysis nominated lipid metabolism as an important biological process that drove sensitivity to ML210. ML210 kills cancer via induction of ferroptosis through covalent interactions with its target, GPX4. Inhibition of GPX4 results in uncontrolled PUFA oxidation leading to ferroptosis [27]. However, there are clear biological determinants of ML210 sensitivity as some cancer cells are exquisitely sensitive while others are ambivalent towards it. Our approach correctly prioritized lipid metabolism as an important determinant of response to GPX4 inhibition. In general, cells with high PUFAs relative to MUFAs are more susceptible to GPX4 inhibition [27, 28]. This trend was also found in the Cancer Cell Line Encyclopedia metabolomics analysis, which demonstrated that the abundance of PUFAs was the most correlated with the genetic dependency on GPX4 [42]. Finally, it is known that some cell lines can protect themselves from lipid ROS by upregulating the lipid saturation pathway [43].

In the context of BRAF inhibition, our approach identified Rac1/cytoskeletal signaling as an important biological process underlying intrinsic drug resistance in cutaneous melanoma with oncogenic BRAF. Rac1 is a Rho family GTPase with diverse signaling properties including cytoskeletal regulation [44]. A mutated version of Rac1, RAC1^P29S^, is a well-described driver of MAPK inhibitor resistance and metastasis in cutaneous melanoma [45–48]. Nevertheless, the Rac1 signaling axis can also drive resistance to MAPK inhibition [49, 50].

Our analysis of PTX-response suggests that inhibiting Akt-signaling may act synergistically with anti-tubulin drugs–additional analysis confirmed significant correlation between two anti-tubulin drugs and two selective Akt inhibitors. Co-targeting Akt and microtubules has been previously proposed [51–53]. Elevation of Akt signaling has also been shown to be positively correlated with PTX response in patients [54]. Here we provide -omics scale evidence that support this therapeutic strategy and the use of Akt pathway activation as a biomarker for PTX response. Our analysis also led us to a previously unreported connection between NOTCH3/PAX8 signaling and drug resistance to PTX.

Consistent with the finding that PAX8 is associated with PTX-resistance, patients with high PAX8 signature had worse overall survival [37]. Previous studies on PTX-resistance in ovarian cancer has implicated a critical role of cell adhesion in driving drug resistance and cell adhesion [55–57]. High expression of cell adhesion related genes was also identified using machine learning approaches to non-responders in patients. Consistent with these findings, both PAX8/MECOM and Notch regulated genes in ovarian epithelial cells enrich for cell-adhesion related pathways [37, 38]. Lastly, a deep learning algorithm developed by another research group also observed Notch signaling as an important predictor of PTX-response [39].

In summary, we developed a machine-learning approach to mine publicly available cancer pharmaco genomics data to generate hypothesis on biological pathways that underlie drug sensitivity. We tested our approach on inhibitors of GPX4, BRAF, and microtubules. Our approach revealed pathways that are consistent with existing knowledge on drug resistance to GPX4 and BRAF inhibition, and which were not detected by standard analysis methods. Furthermore, our PTX analysis informs future studies aimed to enhance the efficacy of anti-tubulin drugs.

## Conclusions

We have developed a machine learning approach to inform the biology underlying cancer drug response. Our approach identified already known biological pathways that contribute to the drug response of ML210 and VEM/Dabrafenib. Our analysis also revealed a potentially novel connection between NOTCH3/PAX8 signaling and PTX drug resistance.

## Supplementary Information

**Additional file 1. Fig S1:** Dotted line at AUC of 9 was the cutoff used to separate sensitive from resistant cancers.

**Additional file 2. Fig S2: A)** Minimum feature ranking for each module. **B**) GO Biological Processes pathway enrichment of genes contained within modules presented in **A**). P-values shown are corrected for multiple hypothesis testing using the Holm-Bonferroni method.

**Additional file 3. Supplementary Table S1.** KEGG pathways that passed pathway activity selection for ML210 analysis.

**Additional file 4. Supplementary Table S2.** Top 20 enriched GO Biological Processes of genes returned by Boruta. P-values shown are corrected for multiple hypothesis testing using the Holm-Bonferroni method.

**Additional file 5. Supplementary Table S3.** Top 20 Enriched GO Biological Processes of t-test derived genes for ML210 analysis. P-values shown are corrected for multiple hypothesis testing using the Holm-Bonferroni method.

**Additional file 6. Supplementary Table S4.** Top 20 Enriched GO Biological Processes enrichment of elastic-net derived genes for ML210 analysis. P-values shown are uncorrected for multiple hypothesis testing.

**Additional file 7. Supplementary Table S5.** KEGG pathways that passed pathway activity selection for BRAFi analysis.

**Additional file 8. Supplementary Table S6.** Top 20 GO Biological Processes enriched in important modules for BRAFi analysis. P-values shown are corrected for multiple hypothesis testing using the Holm-Bonferroni method.

**Additional file 9. Supplementary Table S7.** Top 20 GO Biological Processes enrichment of t-test derived genes for BRAFi analysis. P-values shown are uncorrected for multiple hypothesis testing.

**Additional file 10. Supplementary Table S8.** Top 20 GO Biological Processes enrichment of elastic-net derived genes for BRAFi analysis. P-values shown are corrected for multiple hypothesis testing using the Holm-Bonferroni method.

**Additional file 11. Supplementary Table S9.** Top 20 GO Biological Processes enrichment of t-test derived genes for PTX analysis. P-values shown are corrected for multiple hypothesis testing using the Holm-Bonferroni method.

**Additional file 12. Supplementary Table S10.** Top 20 GO Biological Processes enrichment of elastic-net derived genes for PTX analysis. P-values shown are uncorrected for multiple hypothesis testing.

**Additional file 13. Supplementary Table S11.** Top 20 GO Biological Processes enrichment of t-test derived genes for PTX analysis without blood cancers. P-values shown are uncorrected for multiple hypothesis testing.

**Additional file 14. Supplementary Table S12.** Top 20 GO Biological Processes enrichment of elastic-net derived genes for PTX analysis without blood cancers. P-values shown are uncorrected for multiple hypothesis testing.

## Data Availability

The RMA normalized array gene expression matrix and vemurafenib and dabrafenib drug sensitivities were downloaded from GDSC [58]. Drug sensitivities for ML210 were downloaded from CTRP v2 [59–61]. Yap^On^ genes, namely those in PC1^+^, were obtained from [33]. Akt CMAP pathway signature genes were obtained from [31, 32]. Notch3 overexpression data was obtained from [38]. Gene expression of cancer cell lines for confirmatory analysis was obtained from the Cancer Cell Line Encyclopedia (CCLE) [62]. Lastly, code used to perform the analysis and generate the figures is accessible through Github (https://github.com/eyzhu/cancer_drug_ML_analysis). A guide will be provided to perform analysis on other drugs not assessed here. Requests for data from this study should be directed to Dr. Adam Dupuy (adam-dupuy@uiowa.edu).

## Abbreviations

VEM: Vemurafenib
PTX: Paclitaxel
