# Supplementary figures and images for: Machine learning approach informs biology of cancer drug response
Source: BMC Bioinformatics. 2022 May 17;23:184. doi: 10.1186/s12859-022-04720-z (PMC9112473; doi:10.1186/s12859-022-04720-z)

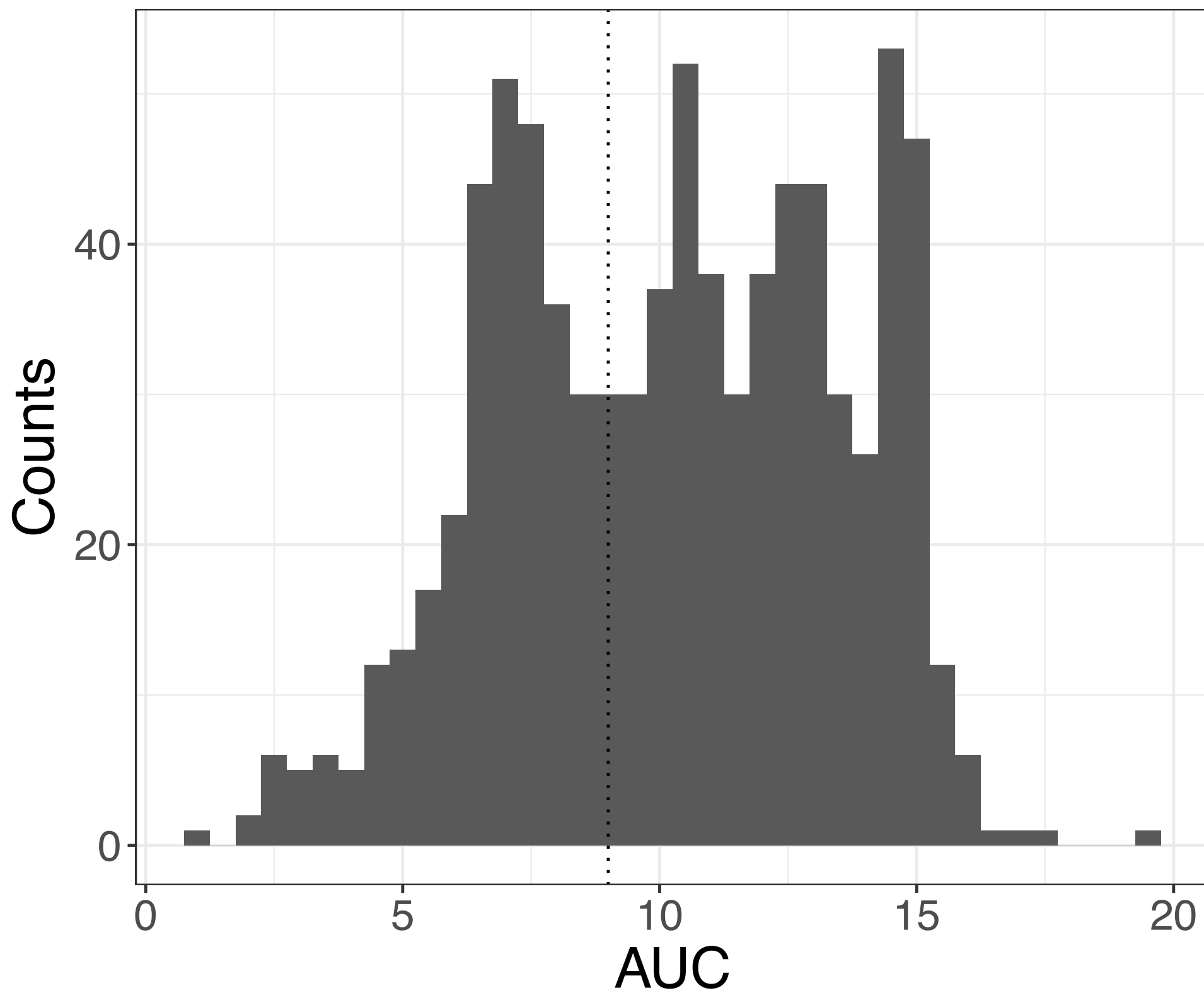

Supplement: Supplementary file 1 — Additional file 1. Fig S1: Dotted line at AUC of 9 was the cutoff used to separate sensitive from resistant cancers. [file 12859_2022_4720_MOESM1_ESM.pdf]

A

Gene

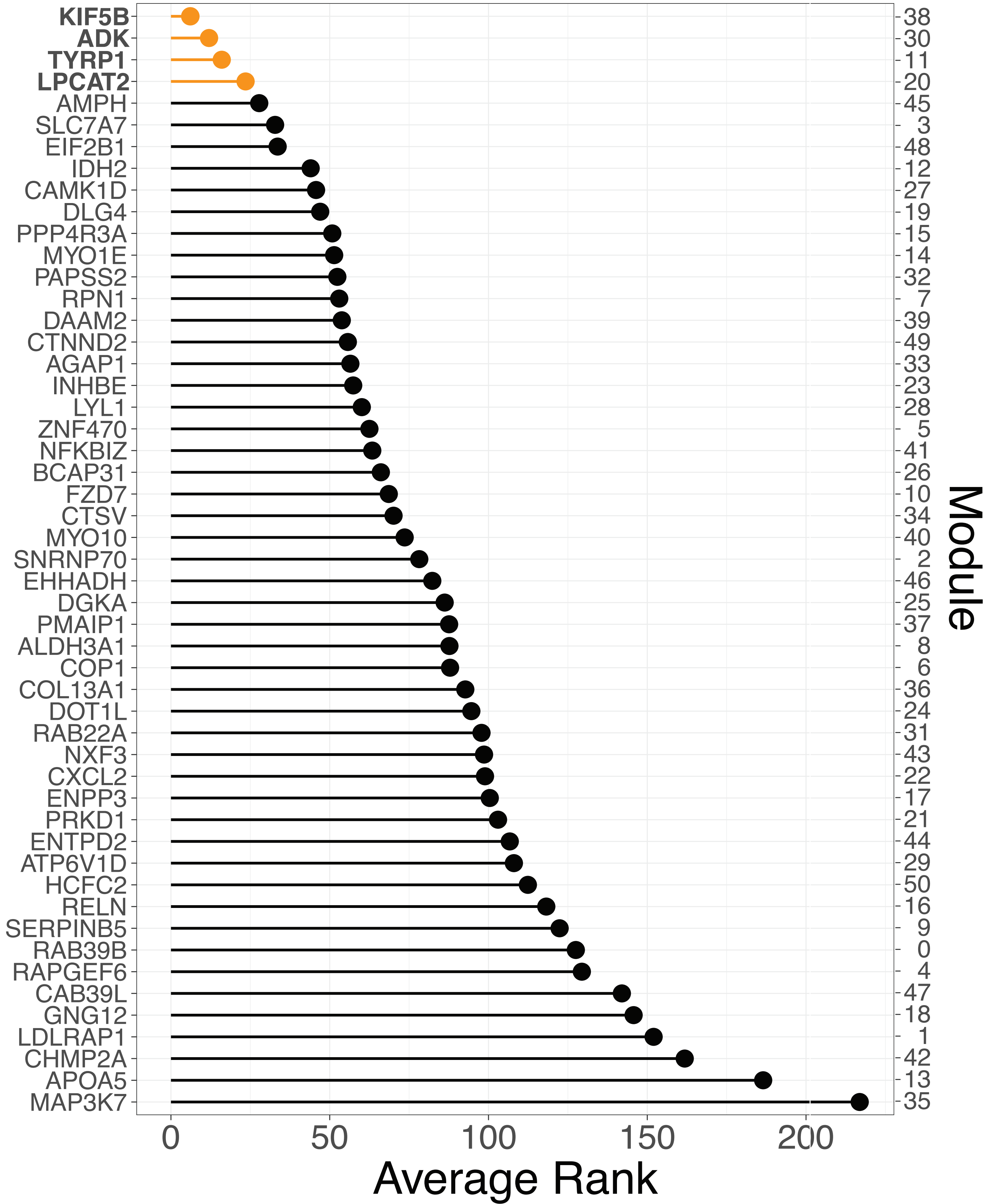

B

Pathway

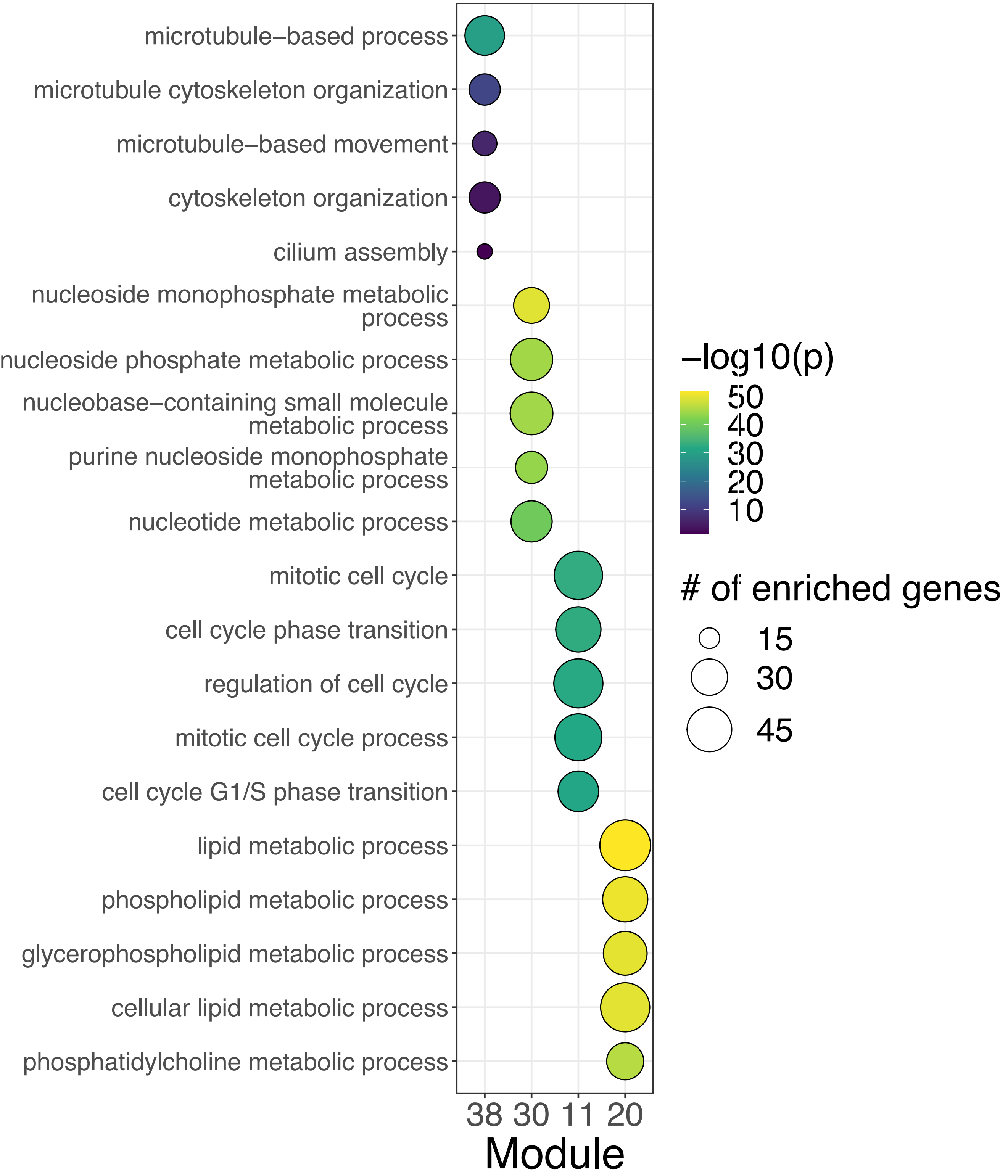

Supplement: Supplementary file 2 — Additional file 2. Fig S2: A) Minimum feature ranking for each module. B) GO Biological Processes pathway enrichment of genes contained within modules presented in A). P-values shown are corrected for multiple hypothesis testing using the Holm-Bonferroni method. [file 12859_2022_4720_MOESM2_ESM.pdf]
